# Assessing Concerns and Care Needs of Expectant Parents: Development and Feasibility of a Structured Interview

**DOI:** 10.3390/ijerph18189585

**Published:** 2021-09-11

**Authors:** Anne van Driessche, Henk F. van Stel, Remy M. Vink, Ingrid I. E. Staal

**Affiliations:** 1 Municipal Health Service Zeeland, 4460 AS Goes, The Netherlands; annevandriessche@live.nl; 2 Julius Centre for Health Sciences and Primary Care, Department of Healthcare Innovation and Evaluation, University Medical Centre Utrecht, 3584 CG Utrecht, The Netherlands; 3 TNO Innovation for Life, 2316 ZL Leiden, The Netherlands; remy.vink@tno.nl

**Keywords:** preventive child health care, expectant parents, risk factors, development, parenting, risk assessment, prevention, prenatal home visit

## Abstract

Many adverse situations for parenting and healthy child development can be detected before a child’s birth. The aim of this project was to develop and test an instrument to use in prenatal home visits, to improve the identification of adverse situations and care needs during pregnancy. The preSPARK is based on a valid and reliable broad-scope structured interview called SPARK (Structured Problem Analysis of Raising Kids). The preSPARK focuses on 12 topics ranging from aspects of the period before pregnancy to future parents’ expectations. The preSPARK was tested in daily practice for feasibility and discriminative capacity. User experience was assessed from the perspective of the professional. In total, 64 home visits using the preSPARK were carried out by 21 nurses. About 24% of the expectant parents needed intensive help or immediate action on one or more topics. The risk assessment showed 29% of the participants were at high risk, 40% at increased risk, and 31% at low risk for future parenting and child developmental problems. The nurses indicated that the preSPARK provides a good structure for home visits and gives insight in interrelated factors. The preSPARK is feasible in daily practice and clarifies risks and care needs of expectant parents.

## 1. Introduction

Many adverse situations for a child healthy development appear to be present before birth [1,2,3,4]. It is well recognized that the absence of social support and a low income have influence on a broad array of health disparities, including low birth weight and preterm births [3]. Similarly, studies found that psychological and psychosocial problems in the expectant mother increase the risk for adverse birth outcomes, such as long-term behavioural and development problems in the child [3,5]. Approximately 10% to 25% of pregnant women worldwide have indicators for depression, and 7% to 18% have symptoms indicating anxiety disorders [6]. This, in combination with other adverse (social) environmental factors, can contribute to the development of psychosocial problems in children [6]. Between 10% and 25% of all children under the age of four encounter varying degrees of problems with respect to parenting or psychological, somatic and social development [7,8,9,10].

A core finding in interdisciplinary research into early childhood development and intervention is that the course of development can be altered in early childhood by effective interventions that change the balance between risk and protection [11]. Moreover, expectant parents appear to be more sensitive to lifestyle changes during pregnancy [12]. Early interventions during child development and the process of development of potential problems have the potential to contribute to long-term health gains for the family [12]. In addition, risk assessment in an earlier stage has financial advantages, compared to costs that would be made later [13,14]. Therefore, starting risk assessment during pregnancy instead of after a child’s birth is relevant. 

The main healthcare provider expectant parents see during their pregnancy is an obstetric care provider. The aim of obstetric care providers is mainly focused on birth in a medical way. However, recently, obstetric care providers in the Netherlands advocate more focus on social factors [15,16]. In 2016, the ‘Standard on Integrated Maternity Care’ was recorded into the register of quality by the Dutch Health Care Institute. Since then, all parties in perinatal care are obligated to implement measures regarding prevention, education and the approach to vulnerable pregnant women [2]. Arranging a home visit around week 34 is listed as the responsibility of the obstetric care provider in the new Standard on Integrated Maternity Care. Referring expectant parents to the preventive child healthcare (PCHC) nurse could be a way for them to adhere to this new standard of care. 

The Dutch preventive child healthcare (PCHC) services have a preventive public health approach that reaches almost all children in the Netherlands from age 0 to 18 [17]. PCHC professionals are specialised in strengthening parental capacity and ensuring an optimal development of the child [18]. In most countries, the early detection of parenting problems and developmental issues is an important part of child healthcare (CHC) [7,19,20]. In the Netherlands, early detection has been one of the key tasks of PCHC services since 2002 [21,22] and are a part of the statutory obligations of PCHC [23]. 

Prenatal home visits by PCHC nurses have already been recommended in professional guidelines for optimal prevention in recent years [6]. However, the provision of prenatal home visits for vulnerable pregnant women by PCHC nurses is expected to come into effect as a legal obligation in January 2022 [24]. The main outcomes of the evaluation carried out during prenatal home visits revealed that clients experienced an added value, because they had the possibility to know the PCHC nurse before birth and, together with the nurse, could review the requirements for an optimal start for and with the baby [12]. PCHC nurses reported it was useful to be able to build trust in their clients and therefore detect potential problems and offer support early. Obstetric care providers argued there was more continuity in care, especially for vulnerable families. The key recommendation from this evaluation was that implementation of prenatal home visits should be stimulated nationally. One of the conclusions was also that there was a need for an instrument or tool to structure home visits and improve the early detection of problems in the prenatal phase [12,25].

A structured instrument, called SPARK (Structured Problem Analysis of Raising Kids), for the early detection of parenting problems and/or psychological, somatic and social development problems in toddlers was developed and studied extensively. This research showed that SPARK is a valid, reliable instrument, feasible to use in practice [26]. The results showed that the structure of the conversation and the involvement of parents’ experiences are essential [27]. In these studies, it was found that most risk factors appeared to be related to the parent(s) rather than to the child [26,27,28,29,30]. Potentially, these factors can be detected before the birth of the child. 

These findings and the need for a structured instrument, as stressed in the evaluation of prenatal home visits, led to the development of a structured interview in the prenatal phase. Similar to the SPARK, this instrument aims to give PCHC nurses the opportunity to combine their clinical judgement with the experiences of the expectant parents. This instrument is called preSPARK, referring to the use of the method of the SPARK in the prenatal phase. Here, we describe first the development of the preSPARK, which aims to contribute to the identification of adverse situations and care needs during pregnancy, and then the initial test of its feasibility and discriminative capacity.

## 2. Methods

### 2.1. Instrument

The preSPARK was adapted from the SPARK18 (Structured Problem Analysis of Raising Kids), a valid and reliable broad-scope structured interview. The SPARK18 is used during a home visit or a visit to a well-baby clinic when the child’s age is 18 months and consists of 16 subject areas. 

SPARK18 proved to be valid and discriminates between groups of children at a high, increased and low risk for parenting and developmental problems in a reliable way. The inter-rater reliability for the overall risk assessment is excellent, with an intraclass correlation (ICC) of 0.92 [28]. Van Stel et al. (2012) showed that SPARK18 was practicable and provided useful information that helped in the choice, together with the parents, of the type of care needed in a family. The overall risk assessment of SPARK18 is the strongest predictor for reports to the Advice and Reporting Centres for Child Abuse and Neglect (ARCAN) and Youth Care Agency (YCA) in the 1.5 years after completing the SPARK (odds ratio of high versus low risk: 16.3 (95% confidence interval: 5.2–50.8). The specificity and negative predictive value of both high and increased risk for a report to ARCAN or YCA were high (high risk: 0.97 and 0.99, increased risk: 0.80 and 0.99) [27]. 

With an interactive and iterative process of testing and feedback between the researchers and a working group of PCHC nurses, an adjusted version called preSPARK was developed, based on SPARK18. The process of discussion, adaptation and subsequent testing in a working group of representatives from six different PCHC organizations was done three times. Adaptations were made to the original instrument regarding the topics and the description of the topics used in the manual but not on the structure and the method of SPARK18. Fewer topics remained. Because the preSPARK is used during pregnancy, topics regarding child development are not yet relevant, e.g., language, speech and thought development of the child. Topics regarding the upbringing and growing up of the child as well as the future of the parents were added. 

After the development of the preSPARK, 41 nurses were trained in using the preSPARK. The training for nurses that were familiar with the SPARK included an introduction to the prenatal SPARK, a manual and the existing e-learning tool for the SPARK. The training for the group that was unfamiliar with the use of SPARK was expanded with group discussions of the manual and a face-to-face training including a learning round of prenatal home visits.

The goal of the preSPARK is to help PCHC nurses to conduct a structured interview with expectant parents, to map the care needs and to come to a mutual agreement about subsequent care. The preSPARK uses a three-step model similar to the SPARK [26]. Step 1: detection of problems and concerns; Step 2: clarifying the characteristics and seriousness of the problems and concerns in a dialogue with the expectant parents; Step 3: analysis and definition of a shared decision on what to do next. The preSPARK examines 12 subject areas (or topics) in the following order: summary of the period before pregnancy; pregnancy experience; health and lifestyle; looking ahead to giving birth; looking ahead to the first days after birth; looking ahead to raising the child; language use of the (expectant) parents; living environment in and outside the home; social contacts and informal support; concerns communicated by others (friends, family, neighbours, healthcare professionals); family issues (for example, health problems, addiction, psychiatric problems, financial problems, divorce or death concerning family members); looking at your (own) future; reflection on whether any topic was forgotten or needed further attention. For an overview of the content of the topics regarding preSPARK with explanatory examples and sample questions that are mentioned in the manual for the PCHC nurses, see Table 1. 

For each topic, the PCHC nurse starts with a short description of the topic making examples and asks the expectant parents if they have experienced any concerns, questions or problems in the last period (Step 1). The seriousness of these concerns can be assessed on a 5-point Likert scale by the expectant parents, ranging from (1), i.e., ‘no concern at all’, to (5), i.e., ‘very concerned’. If concerns are mentioned, the parents are asked to elaborate on the exact nature of the concerns, questions or problems, and whether or not professional and/or informal help—if offered—was sufficient. The discussion of each topic ends with the expectant parents assessing their current perceived need for support on a 6-point Likert scale: (1) no help needed; (2) information wanted; (3) personal advice; (4) counselling; (5) intensive help; (6) immediate intervention required. The PCHC professional makes the same assessment (Step 2). Similar to SPARK18 [27], the information obtained in Steps 1–2 is recorded on a one-page form with a matrix structure: the first column includes all topics, and each of the following separate columns regards a distinct question, i.e., concerns/used support/was the support helpful/current perceived need for support by parents/perceived need for support by nurse. After all the topics have been covered, the PCHC nurse discusses with the parents the amount and content of care needed in the following months (Step 3) and notes this together with a description of the concern or problem on the second page, on which possibilities for further care have been pre-printed. Having done this, the PCHC nurse ends the visit and subsequently makes an overall risk assessment on the third page, assigning the child a low, increased or high risk for parenting problems and development problems in the future. The PCHC nurse bases this overall risk assessment on the information from the interview and on an exam of factors that might positively or negatively influence this risk assessment. This structured elaboration includes the observation of several factors, pre-printed on the third page: health, lifestyle and vulnerability of the pregnant woman; involvement of, and agreement with the father-to-be; growth and development of the unborn child; manifest problems (such as major life events, history of psychiatric illness or financial problems); social support and living environment (hygiene, housing, family composition). Population characteristics are collected in a page added to the preSPARK that contains demographic items. In addition to study purposes, this information is also used by practice to start a file for the PCHC.

### 2.2. Study Design

The goal of the next phase was to test the feasibility and initial test results of the discriminative capacity of the preSPARK in daily practice. If nurses are aware that a vulnerable mother that they already know from previous pregnancies is pregnant again, they can refer these mothers for a prenatal home visit themselves. However, it is most common that obstetric care providers refer potentially vulnerable or vulnerable pregnant women to a PCHC nurse for a prenatal home visit. To identify vulnerable pregnant women, a common definition of frailty drawn up by the municipality of Rotterdam and the Department of Obstetrics and Gynaecology was used [32]. The definition includes varying degrees of vulnerability and is also applied by the new law, mentioned in the introduction [24], that will come into effect from 2022. If the expectant parent(s) agreed, the PCHC nurse contacted them and made an appointment for a prenatal home visit. The home visit started with the structured interview using the preSPARK. The interview was followed by a request (verbal and written) for informed consent to participate in the study and to use the information recorded in the preSPARK for scientific research. 

To assess PCHC nurses’ user experience, we administered to them a short questionnaire used in previous research with the SPARK [28], which in turn was an adapted version of YHC Nurses’ Skills Questionnaire meant for increasing parents’ parenting competences [33]. This questionnaire was slightly adapted to assess PCHC nurses’ skills to increase expectant parents’ parenting competences. Some topics that were included in the questionnaire were: nurses’ ability to comfort expectant parents; which topics were discussed sufficiently and insufficiently and related reasons; whether the nurses felt rushed or relaxed during the conversation. All trained nurses were invited to complete the user experience survey digitally. A final meeting with the working group was organized to discuss the experiences and the outcomes of the user experience. 

Following the criteria of the Medical Ethics Review Committee (METC) of the UMC Utrecht, this research was not subject to ‘the Medical Research Involving Human Subjects Act (WMO)’ [34]. 

### 2.3. Data Analysis

The characteristics of the respondents were analysed with descriptive statistics. Percentages of concerns experienced by parents and the level of support needed according to parents and professionals were calculated. The 6-point assessments of parents and professionals were dichotomized for readability (the category ‘no help needed’ was omitted). To assess the association between the different questions in the preSPARK, Spearman correlations were computed between concerns, perceived need for support and risk assessment by the nurse. Furthermore, correlations with several known demographic risk factors for child abuse and neglect (CAN) were computed: parents unemployed/unable to work; parents’ low education; parents younger than 20 years; parents not speaking Dutch at home; not two-parents household [4,5,35,36,37]. Summary scores for concerns and perceived need for support were computed by summing the scores for all subject areas and dividing the result by the number of areas. For each subject area, we assessed the differences between expectant parents and professionals on the 6-point scale for perceived need of support, using Wilcoxon signed-rank test. Moreover, we assessed discriminative validity by testing differences in expectant parents and family characteristics between the groups at low, increased and high risk as determined by the preSPARK. These between-group differences were assessed with ANOVA or Kruskal–Wallis test, depending on the variable. All analyses were done using SPSS 24 (IBM Corp, Armonk, NY, USA). Differences and correlations were considered to be statistically significant if *p* < 0.05. Furthermore, data from the user experience of the nurses who filled out the questionnaire are described. 

## 3. Results

In the test phase, the preSPARK was administered by 21 nurses of the 41 trained nurses from 6 different PCHC organisations throughout the Netherlands. From July 2014 to March 2015, 64 home visits using the preSPARK were carried out. Of the expectant mothers, five did not want to participate in the study (7.8%), while in nine cases (14.1%), the informed consent was not filled out. This means that data from 50 home visits were used in the analyses. In all home visits, the mother was present, and the conversation was mainly held with her. In 54% of the home visits, the father was also present. Other children from the same family were present in about 8% of the cases. The mean duration of the home visits was 74 minutes (standard deviation (SD) = 22 min), while completing the preSPARK took on average 38 minutes (SD = 20 min). In this group, 73.3% of the families consisted of a two-parent household, while 11.1% was a one-parent household, and 6.7% a shared household. 

**Figure 1:**
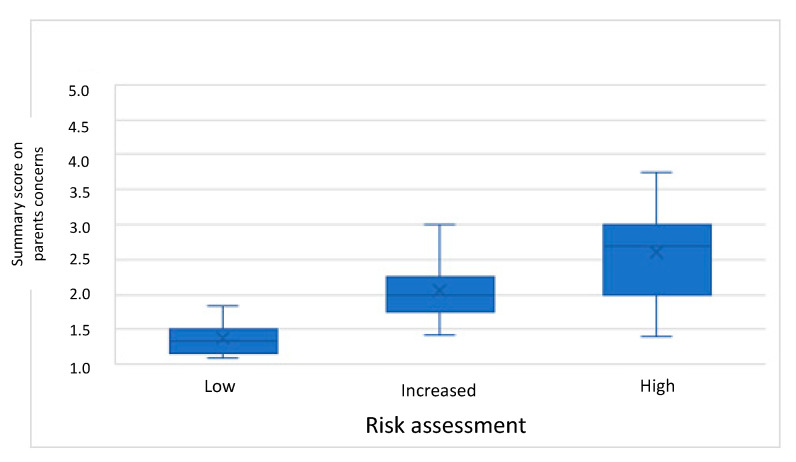
Boxplot of parents’ concerns per risk assessment of the professional.

The first step of the preSPARK is to ask the expectant parent(s) if they have any concerns or problems and whether they experience unfulfilled needs. The median summary score of the topics on experienced concerns by the expectant parents was 1.8 [interquartile range (IQR) = 1.5–2.3; see Figure 1]. 

Almost all expectant parent(s) had concerns, questions or problems in the last period. Topics most mentioned were ‘period before pregnancy’ and ‘family issues’ (see Table 2; first column). 

**Figure 2:**
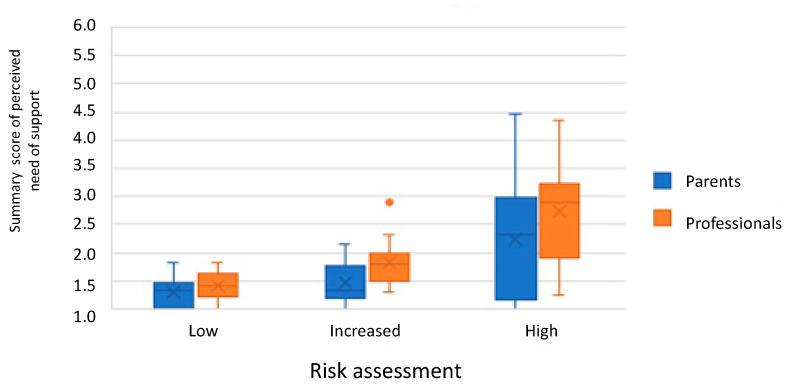
Boxplot of parents’ and professionals’ assessment of need of support per risk assessment of the professional.

The second step of the preSPARK consists in asking both the expectant parent(s) and the PCHC nurse about the current perceived need for support. The median summary score was 1.4 (IQR = 1.1–1.9) for the parents and 1.8 (IQR = 1.4–2.3) for the professionals (see Figure 2). Expectant parent(s) and PCHC nurses mostly agreed on which topics needed further support, but generally the PCHC nurses indicated a higher level of support needed (see Table 2; column 2–5). This occurred mostly for the categories ‘information wanted’, ‘personal advice’ and ‘counselling’, regarding which, the PCHC nurse is able to initiate interventions by him/herself, and was limited for the more serious categories ‘intensive help’ and ‘immediate intervention required’, which require referral to professionals outside PCHC.

Intensive help or immediate action as reported by the professionals was needed by 24% of the expectant parents on one or more topics. Topics with the highest level of support needed were ‘period before pregnancy’, ‘family issues’ and ‘preview of maternity period’, according to the parents. 

The third step of the preSPARK involves an analysis and a decision on what to undertake next. In 31.0% of the cases, follow-up actions were set out before the baby was born, while 23.8% of the parents received regular care. PCHC nurses indicated during the home visit that 38.1% of the cases would need more than regular care after the baby was born. In 7.1% of the cases, a combination of follow-up actions was indicated, such as contact per telephone by the PCHC nurse and referral to HomeStart, a programme for extra support on raising children. In the final step of the preSPARK, the nurse formulates and records an overall risk assessment. The results of this risk assessment indicated that 29% of the participants were at high risk, 40% at increased risk and 31% at low risk for future parenting and developmental problems in children.

The association between the different questions was examined by determining correlation coefficients (Table 3) and by box plots (Figure 1 and Figure 2). Figure 1 shows that a higher risk assessment is associated with an increase in the summary score of concerns reported by the parents. Figure 2 shows an increase in the summary score of perceived need of support by parents and professionals in the different risk assessment scores. The correlation coefficients between the different parts of the preSPARK (concerns, perceived need of support and risk assessment) were moderate to high, varying between 0.53 and 0.80 (see Table 3).

In addition, we explored correlations between the different questions and the known risk factors for child abuse. Significant correlations with known risk factors were found for the following factors: parents unemployed/unemployable, parents’ low education, and a not two-parent household, represented in Table 3. In our sample, there was only one family with the risk factor ‘parents younger than 20 years’. Therefore, no Spearman’s r regarding this risk factor is presented. No significant correlations were found between the overall risk assessment of the professionals and the risk factor ‘parents not speaking Dutch at home’ (Spearman’s r = 0.11). Similarly, no significant correlations were found between these risk factors and parents’ concerns and assessment of need of support from both parents and professionals. 

In Table 4, population characteristics, broken down to clarify risk factors, are presented per risk assessment group. The differences in family composition were significant on a 0.05 level. Furthermore, increased risk and high risk were associated with low education of the mother (*p* < 0.05). 

### User Experience

The survey on user experience was completed by 24 nurses. Three of the respondents did not perform a prenatal home visit in the test phase of the preSPARK. The nurses reported that performing home visits using the preSPARK resulted in more information for the majority of the topics compared to performing the home visits without using the preSPARK. Ten nurses pointed out that several topics were not discussed previously during a home visit without the preSPARK. The preSPARK considerably helped discussing sensitive topics (n = 12). Most nurses indicated that the instrument helped them obtaining more information by illustrating their questions with examples (n = 9) and asking more specifically about parents’ experiences (n = 7). Another advantage was that it was easier for the nurses to link their observations to further questions that broadened their insights (n = 4). 

In total, 19 nurses succeeded in using the structured method of the preSPARK, which gave them reasonable (n = 3) to very much support (n = 15) in the execution of the home visit. Moreover, they indicated that they did not feel or hardly felt rushed (n = 20). The PCHC nurses indicated (n = 21) that the goal of the home visit was (moderately) clear for the majority of the expectant parents. The conversation was pleasant (n = 16) to very pleasant (n = 3), and it was reported that the expectant parents participated actively in the conversation (n = 15). The nurses found that the prenatal home visit using the preSPARK contributes reasonably (n = 12) to significantly (n = 5) to providing insights about the period before pregnancy and birth. 

When asking the PCHC nurses about the added value of the preSPARK, one nurse explained: *‘It offers structure and insight in interrelated factors and it leads you quickly to the primary question or cause.’* With regard to feasibility and applicability, the nurses responded that the use of the preSPARK requires skills, which means that training and gaining experience are essential. Experience with the postnatal SPARK, e.g., SPARK18, helps. The questions are practical, although difficult to ask when there is a language barrier. 

## 4. Discussion

An iterative process was used to develop the preSPARK, an applicable tool for prenatal detection and assessment of future parenting problems and risks for developmental problems in young children. The preSPARK combines the perspectives of the expectant parent(s) and a professional, similar to the SPARK, which is used during a home visit when the child’s age is 18 months [27,28,29]. 

The preSPARK was feasible to use in a selected group referred by obstetric care providers. The percentages of 29% of families classified as at high risk for parenting and developmental problems and 24% as needing help or immediate action on one or more topics are much higher than those found in the general SPARK18 population. As this was a pre-selected group, this is not surprising. Our data indicated that expectant parents think less elaborately about the topics that are not directly important for the very near future but are relevant on the long-term. PCHC nurses can assist the (expectant) parents in anticipating future issues in the long term. This showed that agreement and disagreement between scores of parents and professionals are useful for shared decision making in setting priorities and deciding which follow-up actions to take. The higher numbers we found for unemployment, low education and not two-parent households in the increased- and high-risk groups are in line with existent literature on parent characteristics and risks for children [3,6,38,39]. 

With regard to the training of PCHC nurses in the use the preSPARK, it became clear that the nurses who did not have any experience using the SPARK needed more training and more practice. For nurses who already had experience using the SPARK, the preSPARK was easy to use. Therefore, it was concluded that training is required to use the preSPARK as a supportive aid instead of using it as a questionnaire. This also means that cooperation and coordination with obstetric care providers is necessary to ensure a regular flow of referrals. One of the barriers of using the preSPARK was that it was difficult to apply when there was a language barrier between PCHC nurse and expectant parent(s). However, one can argue that it would be even more difficult to perform a home visit in a setting like that without any structure. This is confirmed by SPARK users who use an interpreter in these situations and indicate that it is precisely the structure of the SPARK that makes the collaboration with the interpreter effective.

Early intervention to prevent adverse outcomes and the effective integration of required services as soon as problems are identified may reduce the prevalence or severity of certain outcomes and will contribute to an effective and efficient use of health resources over the child’s life course [40]. There is ample evidence that the presence of both medical and non-medical risk factors predicts adverse outcomes at birth [41]. Although obstetric care providers have also realised this and shifted their focus from solely medical risks to a social approach, the instruments they use are limited to score cards or questionnaires, often without dialogue [15]. There are several reasons why using the preSPARK method in addition to screening score cards or questionnaires is advantageous in the prenatal health care setting. In general, (expectant) parents are hesitant to fill out score cards, due to ‘information getting on paper’ [6,42]. Another experienced disadvantage of using score cards and questionnaires is that it is less likely for parents to participate in the decision-making process [21]. Additionally, the risk of reporting socially desirable biases on sensitive topics might be higher when using questionnaires only [42], considering PCHC services are sometimes viewed as a detection appliance for child abuse [21]. 

Other prenatal risk assessment instruments applied and tested in PCHC, by which the nurse assesses risks in a broad-structured dialogue with the (expectant) parents while at the same time being able to initiate follow-up actions when needed and building up a relationship with the families the nurse is going to visit and see more often during the child’s development, do not exist to our knowledge. Besides individual risk factors, the accumulation of heterogeneous risk factors is especially important when it comes to adverse health outcomes [41], highlighting the added value and innovativeness of preSPARK home visits. Legally, prenatal action by PCHC nurses is expected to be labelled as obligatory by the Dutch standard task set for PCHC institutions from January 2022 [24], offering opportunities for the preSPARK to prove its added value. 

## 5. Conclusions

This study shows that the preSPARK is a feasible instrument. The preSPARK contributes to high-quality conversations strategies and an open, person-centred attitude. The preSPARK meets the needs of the professional according to the evaluation of prenatal home visits [12,25] and offers an accessible opportunity for early help on a broad scope of topics for vulnerable families. Further studies on reliability, validity, diagnostic accuracy and user experience of all stakeholders, including expectant parents and obstetric care providers, are recommended.

This developmental study has a number of limitations. First, we were able to include only 50 home visits in the analyses. Second, the (discriminative) validity of the preSPARK was only partly assessed. More detailed results on validity and reliability are required. Although the results of the user experience among PCHC nurses were generally positive, it would be useful to gain more knowledge about experiences of expectant parents and obstetric care providers. The strengths of this study are that the preSPARK was developed together with PCHC professionals and that it was tested in daily practice. 

## Figures and Tables

**Table 1:** Content of the topics regarding preSPARK, with explanatory examples and sample questions.

Topics	Main Question(s)	Illustrative Examples (such as e.g., ….)
1. Summary of period before pregnancy	How was your life before you were pregnant?	We first start with reviewing the period before you were pregnant. What was your life like without children? How was your own childhood?
2. Pregnancy experience	How do you like being pregnant?	Mood, reactions from others Is your pregnancy planned, desired? How do the people around you react to the news of your pregnancy? Do you notice any contact with your baby already?
3. Health and lifestyle	How do you think about your own health and lifestyle (now that you are pregnant)? Do you pay attention to that? How do you do that?	Physical growth, healthy diet, exercise, sleep, fever, (chronic or frequent) illnesses, smoking, drinking, drug use How will you integrate these principles after your child has been born/into raising your child? Are there any illnesses that are common in your family?
4. Looking ahead to giving birth	We look forward to the birth	Expectations, feelings, place where to give birth, preparation, pregnancy gym, yoga or course Who would you like near you while giving birth? Has childcare already been arranged for the other children? Have you thought about how you are going to travel to the hospital? And after childbirth?
5. Looking ahead to the first days after birth	What do you expect from the maternity period?	Maternity care, living area, day–evening–night rhythm Have you thought about your preference with regard to your child’s nutrition? What preparation did you undertake? What are your preferences with regard to visits from other people?
6. Looking ahead to raising your child	Do you already have ideas on raising your child?	Crying, regularity, sleeping Are you, as a father and a mother, in agreement on parenting? How was your family upbringing when you were a child? Do you know what to expect from me and my colleagues from the preventive child healthcare services?
7. Language use of (expectant) parents (if relevant)	Which language(s) do you speak at home? Have you thought about what language you are going to speak with your child?	Choice of sthe poken language, bilingual upbringing Do you experience difficulties reading a brochure in Dutch or with filling out forms? What is your experience with talking to healthcare providers?
8. Living environment in and outside the home	Do you have any questions or need information about your living environment and the environment outside your home?	Room and opportunity for the child to play, hygiene and safety at home, housing problems, safety or violence in the neighbourhood, discrimination or cohesion in the neighbourhood Do you like the house and the neighborhood where you live? Is this a kid-friendly neighborhood?
9. Social contacts and informal support	How do you experience the amount and quality of social contacts and informal support? (with respect to your pregnancy and looking ahead to raising your child)	Need for contacts, need for support from others, need someone to share experiences with, need for logistical support Who could you call if you happen to start labor? Do you have any family members that live near you? How is your relationship with them? Does this feel like enough?
10. Concerns communicated by others	Has anybody (for example, your obstetric care provider, employer, parents, parents in law or neighbor) ever indicated any worries? Note: the nurse can also refer to the reason for their visit.	Do you recognize their worries? How did you respond to their worries?
11. Family issues	Sometimes, external factors can influence your expectant child. Such as	Health problems from other family members (are you an informal caregiver?), lifestyle of the (expectant) father, addiction, psychiatric problems, relationship problems, financial problems Are you and the (expectant) father of the child happy together? If unmarried, have you thought about steps to undertake in terms of the legal acknowledgement of your child? Does one of you have a job or do you both work? Have you had any financial consequences from the crisis?
12. Looking at your (own) future	What are your expectations for the future after your child has been born?	Work and/or study, day-care, time for yourself, desire to have more children, role of partner/colleague/friend, sexuality and anticonception * Have you thought about pregnancy/maternity leave? For how long can you take a leave? Will you go back to work afterwards? Have you already thought about how you can fulfill other roles in addition to your mother role?
13. Forgot something?	Do you have any questions or do you need information on a topic that has not been covered during our conversation?	There might be questions about other child(ren) in the family

* If required, the nurse could refer to the national program “Nu Niet Zwanger” (a Dutch program aiming to prevent vulnerable women from becoming pregnant unintentionally) [31].

**Table 2:** Concerns experienced by parents, level of support needed according to parents and professional, per topic.

Topics	Parent Concerns	Perceived Need of Support				*p*-Value *
		Parents Assessment *		Professional Assessment *		
	Concerned/very Concerned (%)	Information Wanted/Personal Advice/Counselling (%)	Intensive Help/Immediate Intervention Required (%)	Information/Personal Advice/Counselling (%)	Intensive Help/Immediate Intervention Required (%)	Parents vs. Professional
Summary of period before pregnancy	31.9	23.9	13.0	34.9	14.0	0.34
Pregnancy experience	10.2	19.6	6.5	34.9	7.0	0.014
Health and lifestyle	6.0	25.5	2.1	43.2	0	0.13
Looking ahead to giving birth	14.3	29.8	6.4	45.7	8.7	0.033
Looking ahead to the first days after birth	14.0	43.5	6.5	57.4	6.4	0.44
Looking ahead to raising your child	2.0	37.0	2.2	73.9	2.2	<0.001
Language use	5.1	25.7	2.9	48.6	2.9	0.009
Living environment (in and outside the home)	14.0	10.6	8.5	23.9	8.7	0.014
Social contacts and informal support	8.2	17.8	2.2	31.8	2.3	0.016
Concerns communicated by others	21.3	21.3	10.6	39.1	10.9	0.004
Family issues	30.6	27.9	9.3	44.2	9.3	0.011
Looking at your (own) future	8.5	7.0	4.7	16.3	7.0	0.034
Forget something?		8.3	0	23.1	0	0.100

* The 6-point assessments of parents and professional were dichotomized for readability; the category ‘no help needed’ was omitted. The comparison using Wilcoxon signed-rank test was on the full 6-point scale.

**Table 3:** Correlation coefficients between parents’ concerns, perceived need of support and overall risk assessment and three known risk factors for child abuse.

	Parent Assessment of Perceived Need of Support	Professional Assessment of Perceived Need of Support	Risk Assessment Professional	Parents with Low Education	Parents Unemployed/Unemployable	Not Two-Parent Household
Parent concerns	0.53 **	0.67 **	0.80 **	0.48 **	0.37 *	0.27
Parent assessment of perceived need of support		0.60 **	0.37 **	0.24	0.14	0.07
Professional assessment of perceived need of support			0.71**	0.30	0.26	0.28
Risk assessment				0.51 **	0.45 **	0.41 **

* Correlation is significant at the 0.05 level (two-tailed). ** Correlation is significant at the 0.01 level (two-tailed).

**Table 4:** Population characteristics per risk group.

	Low-Risk Group N(%)	Increased-Risk Group N(%)	High-Risk Group N(%)	*p*-Value
**Family characteristics**				0.041
Two-parent household	14 (93.3)	14 (77.8)	5 (45.5)	
One-parent household		2 (11.1)	3 (27.3)	
Shared household			3 (27.3)	
Other (foster family/adoption/divorcement/living with grandparents)	1 (6.7)	2 (11.1)	0	
**Parent characteristics**				
Age mother, mean in years (SD)	28 (SD 6.2)	25 (SD 2.8)	29 (SD 6.7)	0.24
Mother age <20 years at birth	0	0	0	
Age father, mean in years (SD)	30 (SD 7.5)	26 (SD 3.0)	30 (SD 4.4)	0.15
Father age <20 years at birth	0	1 (8.3)	0	0.56
Ethnicity: non-Dutch mother	8 (53.3)	6 (31.6)	5 (35.7)	0.42
Ethnicity: non-Dutch father	6 (54.5)	5 (38.5)	4 (50.0)	0.73
Language: non-Dutch used at home by the mother	6 (50.0)	5 (31.3)	4 (40.0)	0.61
Language: non-Dutch used at home by the father	5 (41.7)	3 (21.4)	1 (14.3)	0.36
*Education Mother*				0.048
Low	2 (16.7)	4 (26.7)	7 (63.6)	
Intermediate	6 (50.0)	8 (53.3)	3 (27.3)	
High	4 (33.3)	3 (20.0)	1 (9.1)	
*Education Father*				0.081
Low	4 (30.8)	5 (38.5)	7 (77.8)	
Intermediate	4 (30.8)	5 (38.5)	1 (11.1)	
High	5 (38.5)	3 (23.1)	1 (11.1)	
*Employment Mother*				0.71
Employed	6 (40.0)	2 (11.1)	0	
Unemployed	6 (40.0)	8 (44.4)	7 (58.3)	
Unemployable/unable to work	0	4 (22.2)	2 (16.7)	
*Employment Father*				0.36
Employed	10 (66.7)	14 (82.4)	3 (27.3)	
Unemployed	2 (13.3)	2 (11.8)	5 (45.5)	
Unemployable/unable to work	0	0	3 (37.3)	

## Data Availability

The datasets generated and/or analysed during the current study are available from the corresponding author on reasonable request.
